# Genomic Imbalances Are Confined to Non-Proliferating Cells in Paediatric Patients with Acute Myeloid Leukaemia and a Normal or Incomplete Karyotype

**DOI:** 10.1371/journal.pone.0020607

**Published:** 2011-06-09

**Authors:** Erica Ballabio, Regina Regan, Elisa Garimberti, Jochen Harbott, Jutta Bradtke, Andrea Teigler-Schlegel, Andrea Biondi, Giovanni Cazzaniga, Giovanni Giudici, James S. Wainscoat, Jacqueline Boultwood, Joanna M. Bridger, Samantha J. L. Knight, Sabrina Tosi

**Affiliations:** 1 Centre for Cell and Chromosome Biology and Brunel Institute of Cancer Genetics and Pharmacogenomics, Division of Biosciences, Brunel University, West London, United Kingdom; 2 LRF Molecular Haematology Unit, Nuffield Department of Clinical Laboratory Sciences, John Radcliffe Hospital, Oxford, United Kingdom; 3 NIHR Biomedical Research Centre, Oxford and Wellcome Trust Centre for Human Genetics, Roosevelt Drive, Oxford, United Kingdom; 4 Oncogenetic Laboratory, Department of Paediatric Haematology and Oncology, Justus-Liebig-University, Giessen, Germany; 5 Centro Ricerca Tettamanti, Clinica Pediatrica Università Milano-Bicocca, Monza, Italy; Wellcome Trust Centre for Stem Cell Research, United Kingdom

## Abstract

Leukaemia is often associated with genetic alterations such as translocations, amplifications and deletions, and recurrent chromosome abnormalities are used as markers of diagnostic and prognostic relevance. However, a proportion of acute myeloid leukaemia (AML) cases have an apparently normal karyotype despite comprehensive cytogenetic analysis. Based on conventional cytogenetic analysis of banded chromosomes, we selected a series of 23 paediatric patients with acute myeloid leukaemia and performed whole genome array comparative genome hybridization (aCGH) using DNA samples derived from the same patients. Imbalances involving large chromosomal regions or entire chromosomes were detected by aCGH in seven of the patients studied. Results were validated by fluorescence *in situ* hybridization (FISH) to both interphase nuclei and metaphase chromosomes using appropriate bacterial artificial chromosome (BAC) probes. The majority of these copy number alterations (CNAs) were confirmed by FISH and found to localize to the interphase rather than metaphase nuclei. Furthermore, the proliferative states of the cells analyzed by FISH were tested by immunofluorescence using an antibody against the proliferation marker pKi67. Interestingly, these experiments showed that, in the vast majority of cases, the changes appeared to be confined to interphase nuclei in a non-proliferative status.

## Introduction

Acute myeloid leukaemia is a heterogeneous disease from both clinical and molecular points of view [1]. The majority of AML patients (both paediatric and adult) show specific chromosome rearrangements that can be detected using conventional methods of chromosome banding and the identification of these chromosome abnormalities impacts upon both the diagnosis and prognosis of leukaemia patients [2]–[5].

In the clinical diagnostic setting, conventional cytogenetics by chromosome banding detects chromosomal abnormalities in the majority of AML patients. However, even a decade ago a normal karyotype was observed in a high number of cases, up to approximately 40% of all patients. In paediatric patients, the proportion of cases with no detectable abnormal clone is considered only approximately 20% [2], [6]. This proportion is even lower when considering the fact that a number of submicroscopic genetic alterations have been uncovered. Examples of these are *MLL* partial tandem duplication [7], mutations of the transcription factor *C/EBPα* [8], mutations of the *FLT3* gene [9] and mutation in the NPM1 protein [10].

These submicroscopic changes have an impact on prognosis that challenges the general concept that normal karyotype AML is usually associated with an intermediate prognostic value [2], [11].

The advent of aCGH technology has improved substantially the detection of genomic imbalances in cancer, allowing the identification of new candidate genes [12]. Recently aCGH has identified cryptic copy number alterations (CNAs) in AML patients with complex karyotypes [13], with known chromosomal abnormalities [14] and in 15–16% of adult cases with normal karyotypes [15], [16]. These abnormalities consist of small gains or losses of genomic material ranging in size from 0.2 to 4.1 Mb, with losses usually more common than gains [14]–[16].

To investigate further the role of CNAs in AML, we set out to identify alterations that might have been missed by cytogenetic analysis of banded chromosomes in a cohort of AML patients with reported normal or incomplete karyotypes. We used a genome-wide aCGH platform, with a probe spacing of ∼1 Mb, to analyze DNAs from 23 paediatric AML cases. We performed FISH to both interphase nuclei and metaphase chromosomes to verify the changes found by aCGH. Moreover, in a selected number of patients we investigated the proliferative status of the leukaemic cells to determine whether the chromosomal abnormalities identified might be confined to proliferating or non-proliferating cells.

## Results

### Discovery of large cryptic CNAs in paediatric AML patients with normal karyotypes

**Figure 1:**
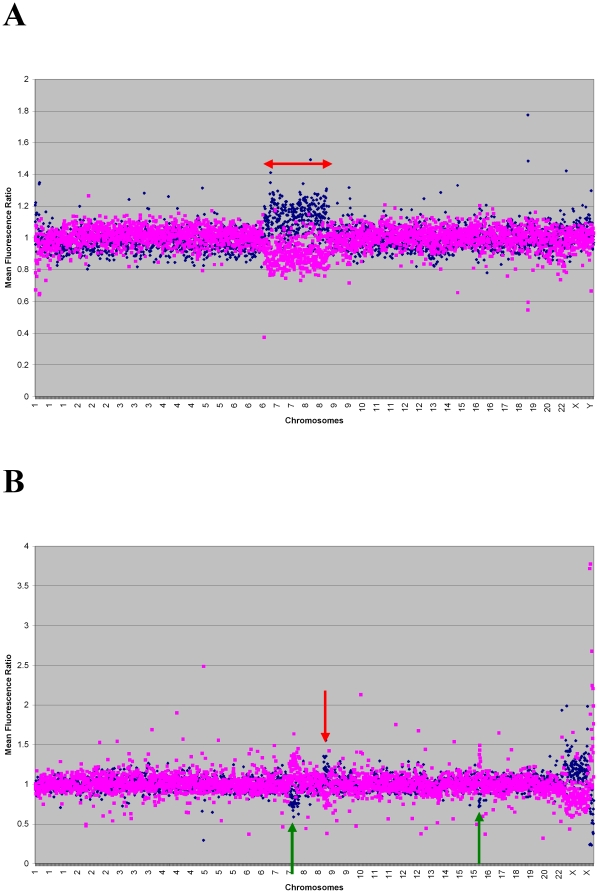
aCGH results of patients no. 1 (A) and 11 (B). On the Y-axis: mean fluorescence ratio; on the X-axis: ordered chromosomal location. Results from the test versus reference and dye-swap experiments are shown in blue and pink, respectively. (A): genomic gains involving chromosomes 7 and 8 are detected in the sample from patient no. 1 and are indicated by the red arrows. The subtle ratio changes are attributable to mosaicism (see FISH results). (B): genomic losses involving 7q and 16p12 are detected in the sample from patient no. 11 and are indicated by the green arrows; a gain involving 8q is indicated by the red arrow.

A total of 23 paediatric patients with AML and reported normal karyotype were analyzed for CNAs by aCGH. The karyotypes had been assessed previously either by Q-banding (patient nos. 1–5) or by G-banding (patient nos. 6–23). A number of 20 metaphases were analyzed for each patients, when possible, before a diagnosis of normal karyotype was made. In patients no. 1–3, 5 and 18, diagnosis of normal karyotype was made based on the analysis of the few metaphases that were retrieved from the samples (<20). The clinical and cytogenetic data for the patients and the aCGH results are documented in Table 1 and described according to the International System for Human Cytogenetic Nomenclature [17]. In total, 15 large CNAs were identified in seven of the 23 paediatric patient samples; gains were detected in six of the seven cases and losses in three cases, the majority involving entire chromosomes or large chromosomal regions. To summarize the aCGH results: three copies of chromosome 7 and three copies of chromosome 8 were detected in patient no. 1; three copies of chromosome 19 were detected in patient no. 2; a gain of 1p36.2p32.3 together with a gain of 11q12.3q13.4 were detected in patient no. 4; a gain of regions 1p36.2p32.3, 9q33.3q34.3, 13q34 and 22q13.1q13.3 were detected in patient 5; a loss of 7q31.2q36.3 and 16p12.2p12.1 together with a gain of 8q24.12q24.3 were detected in patient no. 11; a loss of 15q13.3q21.1 was detected in patient 12 and a loss of 4q35.2 together with a gain of 13q31.1q33.1 were detected in patient no. 19 (Table 1). One of the gains, the 13q34 gain noted in patient no. 5, encompassed a region of polymorphism documented in the Database of Genomic Variants (DGV) and was excluded from follow-up studies. The remaining 16 samples revealed small putative aberrations that were also excluded from the follow-up studies. Examples of the plots showing the CNAs detected in patients no. 1 and no. 11 are shown in Figure 1.

**Table 1:** Clinical and cytogenetic data of the paediatric and adult patients with AML and no reported chromosomal abnormalities analyzed in the present study.

Pt	Age/gender	Disease	Karyotype	no. of metaphases analyzed	% Blasts	Revised abnormalities after aCGH
1	4y/M	AML-M1	46,XY	15	86	arr 7(1–158,821,424)x3,arr 8(1–146,274,826)x3
2	10y/M	AML-M1	46,XY	12	75	arr 19(1–63,811,651)x3
3	0.9mo/F	AML-M5a	46,XX	10	98	
4	12y/F	AML-M5a	46,XX	20	90	arr 1p36.2p32.3(1–53,386,269)x3*,arr11q12.3q13.4(62,515,049–70,795,221)x3*
5	11y/M	AML-M5b	46,XY	10	75	arr 1p36.2p32.3(1–53,386,269)x3*,arr 9q33.3q34.3(127,088,200-141,213,431)x3,arr 13q34(112,590,255-115,169,878)x3,arr 22q13.1q13.3(38,481,453-49,928,069)x3*
6	4y1mo/M	AML-M4	46,XY	31	>90	
7	15y3mo/M	AML-M6	46,XY	32	>90	
8	11y/F	AML-M4	46,XX	20	>90	
9	12y5mo/M	AML	46,XY	33	>90	
10	8mo/F	AML-M7	46,XX	30	>90	
11	16y1mo/F	AML-M2	46,XX	33	>90	arr 7q31.2q36.3(116,864,746-156,509,391)x1,arr 8q24.12q24.3(119,730,121–146,195,298)x3,arr 16p12.2p12.1(21,397,775-26,819,858)x1
12	11y/F	AML-M4	46,XX	29	>90	arr 15q13.3q21.1(31,421,666-49,223,905)x1
13	14y9mo/M	AML-M4	46,XY	29	>90	
14	9y/M	AML	46,XY	34	>90	
15	12y1mo/F	AML	46,XX	45	>90	
16	4y1mo/M	AML-M4	46,XY	38	>90	
17	3y1mo/F	AML-M1	46,XX	25	>90	
18	12y7mo/F	AML	46,XX	12	>90	
19	7y5mo/F	AML	46,XX	31	>90	arr 4q35.2(188,285,250-190,782,221)x1,arr 13q31.1q33.1(85,885,263-104,566,220)x3
20	17y9mo/M	AML-M2	46,XY	34	>90	
21	15y3mo/M	AML	46,XY	34	>90	
22	2y10mo/F	AML	46,XX	36	>90	
23	6y10mo/F	AML-M2	46,XX	23	>90	

*copy number abnormalities not verified by FISH.

### FISH studies

#### Verification of CNAs identified by aCGH

**Figure 2:**
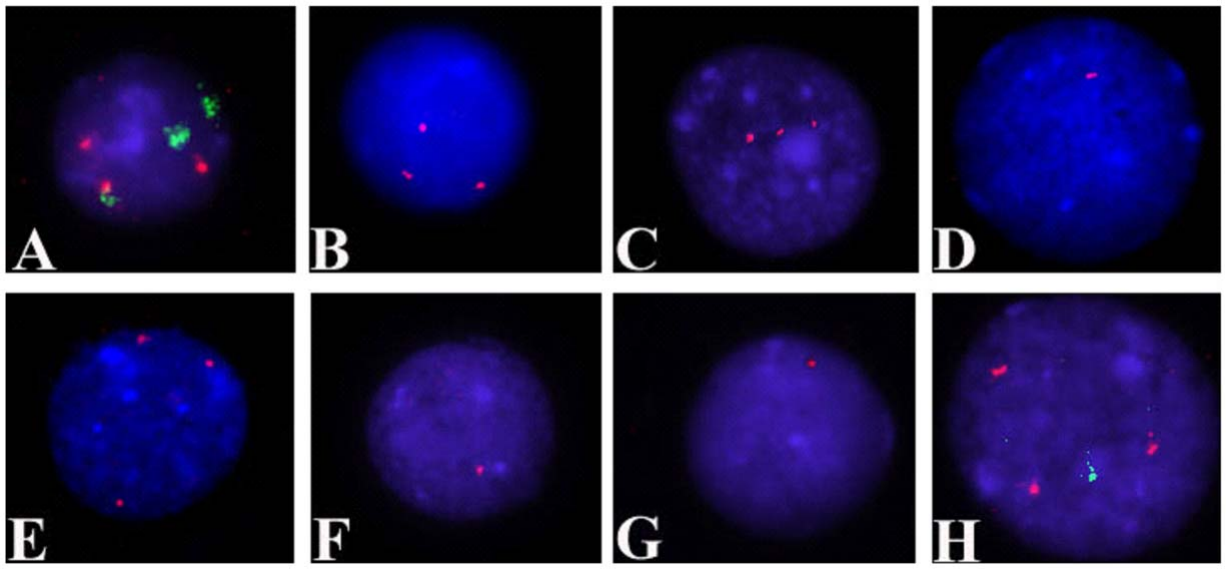
Representative FISH images confirming CNAs in the nuclei of patient samples. (A) Dual-colour FISH signals on nucleus of patient no. 1 confirm trisomy 7 and trisomy 8 in the same clone. Green signals correspond to chromosome 7 centromere, and red signals correspond to chromosome 8 centromere. (B) Trisomy 19 is shown on a nucleus of patient no, 2 using probe RP11-197O4 (hybridization signals in red). (C) Three red signals corresponding to probe RP11-399H11confirm the presence of three copies of 9q in patient no. 5. (D, E and F) FISH performed on patient no. 11 confirmed: (D) a monosomy of 7q22 as shown by the presence of one red hybridization signal corresponding to probe RP11-193I7; (E) a trisomy of 8q24.13 as shown by the presence of three red signals corresponding to probe RP11-16G11 and (F) a monosomy of 16p12 as shown by one red signals corresponding to probe CTD-2515A14. (G) In patient no. 12, monosomy of 15q21.1 is shown by one red signal corresponding to probe RP11-485O10 (H) Dual-colour FISH on a nucleus from patient no. 19 confirms the presence of a trisomy for 13q32.2 (three red signals corresponding to probe RP11-383H17) and monosomy of 4q35.2 (one green signal corresponding to RP11-45F23) in the same leukaemic clone.

Ten of the putative CNAs identified by aCGH were investigated further by FISH, using probes specifically targeted to the chromosomes involved in the abnormalities. Importantly, the cell preparations used for FISH were derived from samples obtained at the same time as those used for DNA extraction for the aCGH experiments. The BAC clones used for FISH are listed in Table 2 together with a summary of the results showing that ten putative CNAs were verified by FISH. Examples of FISH images obtained using samples from patients no. 1, 2, 5, 11, 12 and 19 are shown in Figure 2.

**Table 2:** Summary of FISH results for all AML paediatric patients reported in this study.

Pt	Chr	Probe	Genomic Location (hg35)	Number of metaphases observed by FISH	Percentage of interphase cells showing CNAs*				Co-existence of CNAs involving different genomic regions in the same clone
					Single copy loss	Gain of an extra copy	Gain of two extra copies	Gain of three extra copies	
1	7	p7t.1	7 cen	4	0.0	40.9 (4.8±6.8)	1.8 (0±0)	0.0	Yes: confirmed by FISH
	8	D8Z2	8 cen	3	0.0	26.4 (1.3±0.6)	0	0.0	
2	19	RP11-197O4	19p13.2 (10,509,359-10,680,459)	0	0.0	48.5 (4.7±6.6)	2.9	0.0	ND
5	9	RP11-399H11	9q34.3 (131,495,347-131,716,718)	2	0.0	14.4 (2.8±3.4)	0.0	0.0	ND
11	7	RP11-193I7	7q32.1 (130,884,337-131,060,545)	0	80.1 (2.8±3.4)	0.0	0.0	0.0	ND
	8	RP11-16G11	8q24.13 (122,320,844-122,475,611)	0	0.0	66.0 (3.8±0.8)	0.0	0.0	
	16	CTD2515A14	16p12 (24,657,417-24,876,429)	1	79.9 (3.8±1.2)	0.0	0.0	0.0	
12	15	RP11-485O10	15q21.1 (46,628,904-46,803,043)	0	28.0 (2.9±2.4)	0.0	0.0	0.0	ND
19	4	RP11-45F23	4q35.2 (190,867,466-191,015,883)	0	66.8 (2.7±3.4)	0.0	0.0	0.0	Yes: confirmed by FISH
	13	RP11-383H17	13q32.2 (97,344,722-97,505,793)	0	0.0	55.1 (3.9±5.0)	14.0 (0.6±0.6)	0.9 (0±0)	

*The percentage of cells presenting CNAs is indicated, taking into account the cut-off levels established using normal controls. In brackets: cut off levels (mean % of control ±2x st dev). The values shown are the result of the FISH analysis performed on at least 200 nuclei per patient. ND = not done.

#### Cell to cell pattern of mosaicism

For all of the 10 CNAs verified by FISH, the FISH analyses also revealed that the cell population assayed was mosaic for the abnormalities. Furthermore, the CNAs appeared to be confined to the interphase nuclei and were not observed in any of the metaphases noted during this set of analysis (Figure S1). In two cases, patient no. 1 and no. 19, who had more than one CNA, dual-colour FISH experiments were carried out to investigate whether the CNAs were occurring within the same interphase cells, or whether they were differentially distributed across cells (Figures 2A and 2H, respectively). For patient no. 1, 14/50 (28%) of the interphase nuclei gave 3 red signals for chromosome 7 and 3 green signals for chromosome 8 in the same nucleus, whereas only 3/50 (6%) of interphase nuclei appeared to carry only a single extra chromosome 8. For patient no. 19, 92/152 (60.5%) interphase nuclei gave three green signals for chromosome 13 and one red signal for chromosome 4 occurring within the same interphase cells.

#### Proliferation experiment

In order to determine if the interphase cells carrying the chromosomal abnormalities were proliferating or non-proliferating/quiescent, immunofluorescence with an antibody specific for the proliferation marker pKi-67 was performed in combination with FISH. Anti-pKi-67 antibodies decorate the nucleolus in proliferating cells and also associate with heterochromatic regions in G1 cells [18], [19]. In cases where more than one CNA was present, only one representative FISH probe, randomly selected, was used. A total of four paediatric patient samples were tested in this way and in each case at least 200 interphase cells were scored. The results described in Table 3 show that, for each case, the proliferative state of the cells carrying the CNA was different from that of the cells that were not carrying the CNA. For the CNA cells, the average of non-proliferating (pKi-67 negative) cells was 96.75% (based on 4 patient samples ranging from 94–100%) compared with an average of 72.25% of cells that did not carry CNAs (based on 4 patient samples ranging from 69–75%). For all patients, the difference in cell percentages between the non-proliferating cells without CNAs (2 signals) and the non-proliferating cells with CNAs (3 signals) is statistically significant. Indeed, if a one-tailed hypothesis test is performed on the difference in population proportions, then the result is significant even at 99% confidence.

**Table 3:** Summary of combined immunofluorescence and FISH results for 4 paediatric patients.

		% Nuclei with 2 signals (absolute number of nuclei)		% Nuclei with 3 signals (absolute number of nuclei)	
Pt no.	Chromosome	Ki-67 positive	Ki-67 negative	Ki-67 positive	Ki-67 negative
1	7	26 (58)	74 (170)	2 (3)	98 (118)
2	19	25 (32)	75 (98)	6 (5)	94 (68)
5	9	29 (45)	71 (110)	5 (2)	95 (38)
11	8	31 (24)	69 (55)	0 (0)	100 (101)

**Figure 3:**
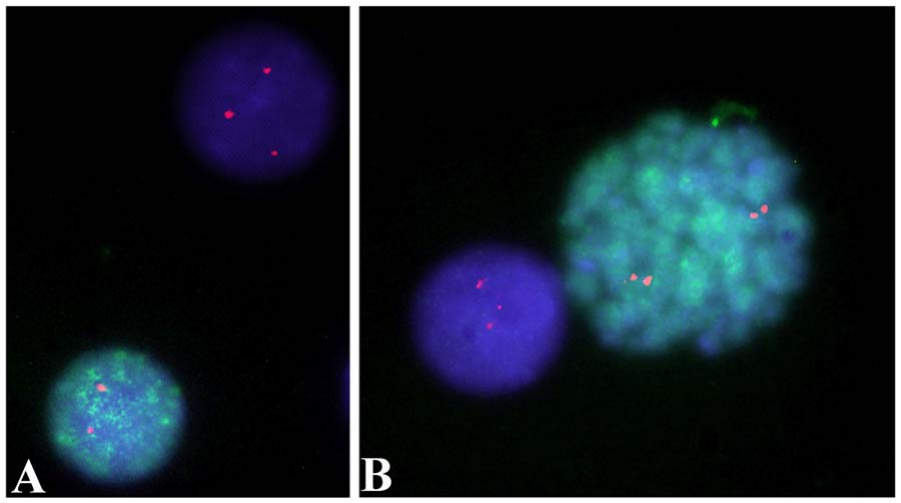
Example of Immuno-FISH performed on cells from patient no. 2. Anti-pKi-67 antibody is shown in green, whereas hybridization signals corresponding to the chromosome 19 specific probe RP11-197O4 is shown in red. (A): the nucleus with two FISH signals is pKi-67 positive, whereas the nucleus with three FISH signals is pKi-67 negative (B): the metaphase with two FISH signals is pKi-67 positive, whereas the nucleus with three FISH signals is pKi-67 negative.

Importantly, during these experiments at least 3 metaphases per sample were also identified and scored. As expected, all were positive for pKi-67, since pKi-67 forms a coat around metaphase chromosomes [19]. However, in contrast to the previous FISH experiments where no abnormalities were observed in the metaphase cells analyzed, two of the six metaphases analyzed in patient no. 1 were this time observed to carry trisomy 7 (Figure 3). Thus, including the original FISH results where seven metaphases could be scored, a total of two out of thirteen (15%) metaphases scored for patient no. 1 carried trisomy 7.

## Discussion

In this study, we tested, by aCGH, a series of paediatric AML patient samples with normal karyotypes to assess the contribution of cryptic CNAs in this cohort. The definition of normal karyotype was based on the analysis of at least 20 metaphases in the majority of cases. It should be noted that in five cases only 10–15 metaphases were analyzed and in three of these cases CNAs were detected. However, in the UK 10–19, metaphases with no chromosomal abnormalities still mandates a normal karyotype result, albeit with a statement about low level clones. Clinically these patients would all be in the same prognostic group. The current recommendations for standardization of criteria in AML, include that at least 20 metaphases are required for the assessment of a normal karyotype [20]. However, it should be noted that the samples reported in this study were analysed prior to the current guidelines (2003–2006), when the criteria were still unclear. Furthermore, in four cases CNAs were found in patients where more than 20 metaphases had been analysed.

To understand the biological origin of the CNAs, we undertook further characterization using FISH and cell proliferation tests. Of the 23 paediatric patients tested, we identified and confirmed large CNAs in seven cases (30%), three of whom carried more than one abnormality.

In these cases the CNAs were noted as large because they encompassed at least one chromosomal band. CNAs that were defined by less than three contiguous clones were generally excluded.

For the remaining 10 CNAs that were verified by FISH, all except the 4q35.2 loss (patient no. 19) were in chromosomal regions that have been reported previously (either wholly or partly) in patients with AML.

Furthermore, for the 10 CNAs that were verified by FISH, a mosaic pattern of hybridization was noted and most significantly, the CNAs were discovered to be confined to the interphase nuclei in all but one case. In this case (patient no. 1), the combined FISH and indirect immunofluorescence assay showed changes in two of the 13 metaphases that could be analyzed.

These results gave an important insight into why the CNAs were likely to have been missed when the patients' chromosome preparations were assessed by routine cytogenetics: karyotyping involves only metaphase cell analyses and in the samples tested, the abnormalities were simply not present in the metaphase cells that could be analyzed. Other reasons for the discrepancy between the karyotyping and aCGH data might include the poor quality of metaphases or the loss of the aberrant clone during the *in vitro* preparation, but given our observations, these seem less plausible. Importantly, the DNA used for the aCGH was extracted from cells obtained at the same time as those used for the karyotyping and FISH experiments and therefore the possibility of clonal evolution can also be ruled out. It could be argued that although the cells were obtained at the same time, those used for aCGH were frozen immediately and then processed for DNA extraction, whereas the ones used for karyotyping were cultured. However, the culture times were never longer than 48 hours, with approximately 50% of the samples being processed after 24 hours of culture. It is unlikely that the leukaemic clone/s would have evolved in such a limited time. It is though possible that the use of a suboptimal medium could result in the selective loss of tumour cells and the cells analyzed in classical cytogenetics are only normal surviving cells.

The observation that the CNAs appeared to occur only in interphase nuclei in six out of the seven patient samples followed up by FISH prompted us to investigate whether this was related to the proliferative status of the cells,. The proliferation study, using anti-pKi-67, demonstrated that the majority (94–100%) of nuclei carrying the abnormality were also negative for the proliferation marker and were therefore in a non-proliferative state. On the other hand, only 69–75% of the nuclei without imbalances were anti-pKi-67 negative. We have shown that the proportion of non-dividing cells carrying the CNAs were significantly higher (at 99% confidence in a one tailed test) than the percentage of non-dividing cells karyotypically normal in each sample. Interestingly, in patient no. 1, where two of the 13 metaphases analyzed presented with an extra chromosome 7, both nuclei were pKi-67 positive as expected. It is possible that these represent slowly-dividing cells rather than cells actively proliferating at a normal rate, therefore could not be identified by chromosome banding analysis. It might be relevant to note that the original cytogenetic analysis on this patient was performed on 15 metaphases, therefore the abnormal clone might have been missed due to the relatively low number of cells analysed in this case. Perhaps increasing the culture time from the conventional 24–48 hours would favour the recruitment of a higher number of metaphases showing the abnormalities, allowing the leukaemic “slowly dividing” cells to reach cell division.

The detection of CNAs in AML with reported normal karyotype is not a novel finding and our data are comparable to those of Armengol *et al.* [21] who identified by aCGH large genomic imbalances in two out of 16 cases of childhood AML, although only three patients of this series had a normal karyotypes. In a previous study, Tyybakinoja *et al* [16] also identified cryptic copy number changes in 15% of adult AML cases with reported normal karyotype. CNAs undetected by G-banding have been revealed by Karst *et al.* [22] not only in normal karyotype AML, but also in AML patients with reported monosomy 7 or trisomy 8 in a total of 34% of cases. Furthermore, since the introduction of interphase FISH to complement the cytogenetic analysis in diagnostic laboratories in the early 1990s, discrepancies between karyotypes and observations of cell nuclei have been reported. This has often been attributed to the analyses being conducted on two different cell populations, dividing versus non-dividing, the assumption being that interphase FISH was performed on non-dividing cells. Following this assumption, Karst *et al* [22] came to the conclusion that the chromosomal aberrations they detected in the AML patients were restricted to the *in vitro* non-proliferating population of tumor cells. However, for the first time, our study has investigated the proliferating status of leukemic cells carrying the CNAs. Using indirect immunofluorescence to reveal the Ki67 proliferation marker, we have generated experimental evidence to support the hypothesis that cryptic CNAs are largely confined to either quiescent or senescent cells.

Clinically, one key question is whether the CNAs identified in the patients with apparently normal karyotype and the observation that they occur predominantly in cells that are not actively proliferating confers any additional diagnostic or prognostic value when evaluating AML patients. In other words, is it clinically relevant that non-proliferating cells have chromosomal aberrations? Further studies, beyond the scope of this report should be aimed at investigating these aspects of AML biology. Traditionally, the prognostic value of a normal karyotype in AML cases has been considered intermediate [2], [4]. However, the ability to detect cryptic abnormalities has indicated that such assertions drawn from a reported normal karyotype, are not always correct. For example, patients who carry a cryptic partial duplication of the *MLL* gene (11q23.3) have an unfavourable prognosis whereas those with mutations of the transcription factor *C/EBPα* (19q12.1) are usually associated with favourable clinical outcome [7], [8]. Mutations of the *FLT3* gene (13q12.2) are again poor prognostic factor whereas patients with a mutation in the *NPM1* gene (5q35.1) as the sole abnormality have a better prognosis [9], [23]. In the patient cohort reported in the current study, the CNAs identified did not harbour any of these prognostic marker genes and there was no clinical outcome data available that would allow conclusive links to prognosis to be made. The prognostic value of CNAs detected by aCGH is supported by the study by Armengol *et al*. [21], who made a general conclusion that four or more genomic imbalances correlated with poor patient outcome in those with a normal karyotype. Furthermore, it should be noted that in clinical practice a copy number alteration equal or above the FISH cut-off of 6% is believed to have an impact [24], [25].

Our original observation that CNAs in patients with normal karyotypes occur predominantly in cells that are not actively proliferating should be taken in consideration when a correlation with clinical outcome is attempted. Furthermore, our aCGH assay, combined with interphase FISH, has enabled us to detect a range of cells with various degrees of aneuploidy within the same sample (chromosome 7 in patient no. 1, chromosome 19 in patient no. 2 and chromosome 13 in patient no. 19), clearly showing evolution of leukemic clones. The proliferation test was performed on two of these samples (patient nos. 1 and 2) and showed that the aneuploidies were largely, but not only, confined to the pKi-67 negative cells in these cases. This implies that it is still possible for that small percentage of pKi-67 positive cells to evolve with respect to the acquisition of additional changes.

Our aCGH system at ∼3 Mb resolution might be considered now obsolete, since higher resolution platforms are continuously made available and are currently being introduced in the laboratory practice. Nevertheless, our system was sufficient to reveal large genomic gains or losses. However, the prognostic value of these findings and the possible impact in the clinical practice needs to be addressed.

Our data may also offer some practical information in terms of clinical laboratory evaluation of leukaemia cases: (i) longer culture times might improve the detection of abnormalities in slowly dividing cells; (ii) aCGH could be considered as a routine tool alongside optimized karyotyping or targeted FISH for the evaluation of AML cases.

aCGH cannot be a replacement for the cytogenetic approaches in AML because it cannot detect truly balanced rearrangements or give positional information. However, it is a powerful tool for the detection of cryptic unbalances that may be missed cytogenetically.

In summary, our study has added to a growing body of evidence showing that a significant proportion of patients with AML harbour cryptic CNAs. Importantly, we have also shown that these CNAs are mostly confined to interphase cells. The novelty of our study resides in the finding that these interphase cells are in quiescent or senescent status. Our findings will need to be exploited further not only towards the establishment of more suitable diagnostic tools, but also towards a better understanding of the biology of AML with apparently normal cytogenetics.

## Materials and Methods

### Patients and ethics statement

Bone marrow samples from 23 paediatric patients with AML, all with normal karyotypes, were used for the aCGH study. A single control DNA sample from the peripheral blood lymphocytes of a healthy subject (sex matched) was routinely used in the lab for aCGH experiments. Three different control samples from peripheral blood lymphocytes of healthy individuals were used for FISH. Clinical and cytogenetic details of all the patients analyzed in this study are summarized in Table 1. Patient nos. 1–5 were contributed by the Paediatric Clinic, San Gerardo Hospital, Monza, Italy. Patient nos. 6–23 were contributed by the Oncogenetic Laboratory, Children's University Hospital, Giessen, Germany. Informed consent for the use of blood or bone marrow samples for research purposes was obtained from all individuals or their parents. Ethical approval to work on these samples was obtained by the Brunel Ethics Committee.

### aCGH protocol

Genomic DNA was extracted using the PUREGENE™ DNA isolation kit (Gentra System, Minneapolis, USA), according to manufacturer's instructions. Reference and test genomic DNAs were sonicated and purified using microcon columns (Millipore, Watford, UK), according to manufacturer's instructions. 2 µg of each purified DNA was labelled overnight, with the appropriate cyanine dye, Cy3dCTP or Cy5dCTP (Amersham Pharmacia, GE Healthcare, Buckinghamshire, UK), using the BioPrime DNA labelling kit (Invitrogen, Inc., Carlsbad, California, USA). Two aCGH tests were performed for each patient: in the first, the patient genomic DNA was labelled with Cy5 dye and the normal DNA reference with Cy3 dye; in the second, a “dye-swap” experiment was carried out where the patient sample was labelled with Cy3 dye and the normal reference with Cy5 dye. Labelled samples from the test and reference samples were combined and hybridized to 1 Mb density, large-insert clone arrays prepared at The Wellcome Trust Centre for Human Genetics, Oxford, UK using the 1 Mb clone set obtained from The Wellcome Trust Sanger Institute (Cambridge, UK), as previously described [26].

The fluorescence intensity data were collected by scanning the arrays in a GenePix 4000B scanner (Molecular Devices, USA), using GenePix Pro 4.1 software (Axon Laboratories). The data were stored in a GenePix Results file (GRP) and imported into Microsoft Excel. A series of customized Macro files were used in Excel. The fluorescence intensity of the local background was subtracted from the raw intensity of each spot. The reference versus test DNA spot intensity ratio was calculated (i.e. Cy5/Cy3) and the median of all the spot ratios within each block was calculated. Normalization of each spot intensity ratio was achieved by dividing the intensity ratio by the median of all spot ratios within each block. For each clone, the mean and standard deviation (SD) across the identical triplicate were calculated. Any spot with a SD>0.2 was discarded from the analysis. If two of the triplicate spots representing a clone had a SD>0.2, the clone was discarded from the analysis. The mean normalized fluorescent intensity ratios were plotted in chart form, from which clones representing gains and losses of chromosomal material were identified.

### Definition of the extent of CNAs

CNAs were detected visually using lower and upper threshold values of 0.8 and 1.2 to indicate loss and gain respectively. These values were calculated using the global thresholding method previously described [26], [27]. We would consider at least three contiguous probes above/below the threshold, that would correspond to a genomic fragment of at least 2 Mb in size.

### FISH analysis

FISH was performed on archival fixed cell and chromosome suspensions prepared from the bone marrow of the patients and from the peripheral blood of the healthy individuals. These same archival suspensions were used previously for the chromosome analysis that defined the karyotype in all patients analyzed. The bone marrow cells used for these analyses were typically cultured in RPMI-1640 medium with 20% foetal calf serum for 24 hours (patient nos. 6, 9, 10, 19–21) or 48 hours (patient nos 1–5, 7, 8, 11–18, 22, 23) prior to harvesting, whereas the peripheral blood cultures were set up for 72 hours prior to harvesting according to existing protocols [28]. Briefly, colcemid is added 30 minutes prior to harvesting in all cases, samples are then treated with hypotonic solution for 15–30 minutes, fixation follows using the standard solution (three parts of methanol and one part of acetic acid). Cells and chromosomes suspensions are then dropped onto clean microscope slides and used for chromosome banding or FISH. The cytogenetic analysis was carried out in two different laboratories: (i) Centro Ricerca Tettamanti, Paediatric Clinic, San Gerardo Hospital, Monza, Italy (patient nos 1–5) and (ii) Oncogenetic Laboratory, Children's University Hospital, Giessen, Germany (patient nos. 6–23). The karyotypes were not reviewed centrally, however they were assessed and checked by two experienced cytogeneticists in each centre. Details of the FISH probes used are given in Table 2. Probes were labelled by nick translation using either biotin-16-dUTP or digoxigenin-11-dUTP and hybridized as previously described [29] Dual colour detection was obtained using Texas red conjugated avidin and anti-digoxigenin FITC conjugated antibodies. Microscope analysis was performed using fluorescence microscope Zeiss Axioplan2 Imaging equipped with specific band pass filter wheel and Sensys cooled CCD camera. Image capture and enhancement was performed using Smart Capture 2 software (Digital Scientific, Cambridge, UK).

### Interphase FISH: Calculation of cut-off values and statistical analysis

CNAs in patients samples were established after comparison with interphase FISH performed on normal controls according to methods published previously [30]. Briefly, a monosomy or a trisomy is assessed when the percentage of cells with one or three FISH signals is greater than the mean % value of the controls ±2 SD. A minimum of 200 nuclei were counted in each sample (both normal controls and patient samples).

### Indirect Immunofluorescence

FISH slides were washed with PBS and monoclonal anti-pKi-67 (Novacastra, Newcastle Upon Tyne, UK) was applied to the slides for 30 min at 37°C. Secondary swine anti-rabbit antibody (DAKO, Denmark), conjugated to fluorescein isothiocyanate (FITC) was then applied to the slides for 30 min at 37°C. Images were captured using a Zeiss microscope. A minimum of 200 nuclei were analyzed in each sample.

### Proliferation test: statistical analysis

We chose to use the hypothesis test on the difference in population proportions [31]. This statistical test is used to determine whether the difference between two proportions is significant. Four different patients were considered. For each patient, the proportion of pKi-67 negative cells was compared between the population of nuclei with two signals and the population of nuclei with three signals. We showed that, for every patient, the proportion of pKi-67 negative cells is statistically higher in the population of nuclei with three signals. The application of the hypothesis test applied to our datasets can be viewed in table S1.

## Supporting Information

Figure S1 Examples of FISH using a centromeric probe specific for chromosome 7 (7cen) on patient no. 1. Two red signals are clearly visible in the metaphase, whereas three fluorescent signals are present in the nucleus in the same sample.
(TIF)

Table S1 Using a one-sided hypothesis test, with a null hypothesis that p_1_ = p_2_, and an alternative hypothesis that p_1_<p_2_, at 99% confidence, the critical test statistic is 2.33. Since the test statistics above are all greater than 2.33, then we can reject the null hypothesis in all cases. Note: it is often recommended that this test only be applied when both the number of successes and the number of failures is at least 5 in both populations. Although this is strictly not satisfied in population 2 above, it is satisfied for the cell population as a whole. Also, if the number of failures in population 2 (i.e. the number of Ki-67 positive cells with 3 signals) was 5 for all patients (a result which is clearly not as significant as the result above), then the result would still be statistically significant at 99% confidence for all patients except patient 5. For patient 5 it would be statistically significant at 98% confidence.
(DOC)
