# Supplementary figures and images for: Genomic Imbalances Are Confined to Non-Proliferating Cells in Paediatric Patients with Acute Myeloid Leukaemia and a Normal or Incomplete Karyotype
Source: PLoS One. 2011 Jun 9;6(6):e20607. doi: 10.1371/journal.pone.0020607 (PMC3111408; doi:10.1371/journal.pone.0020607)

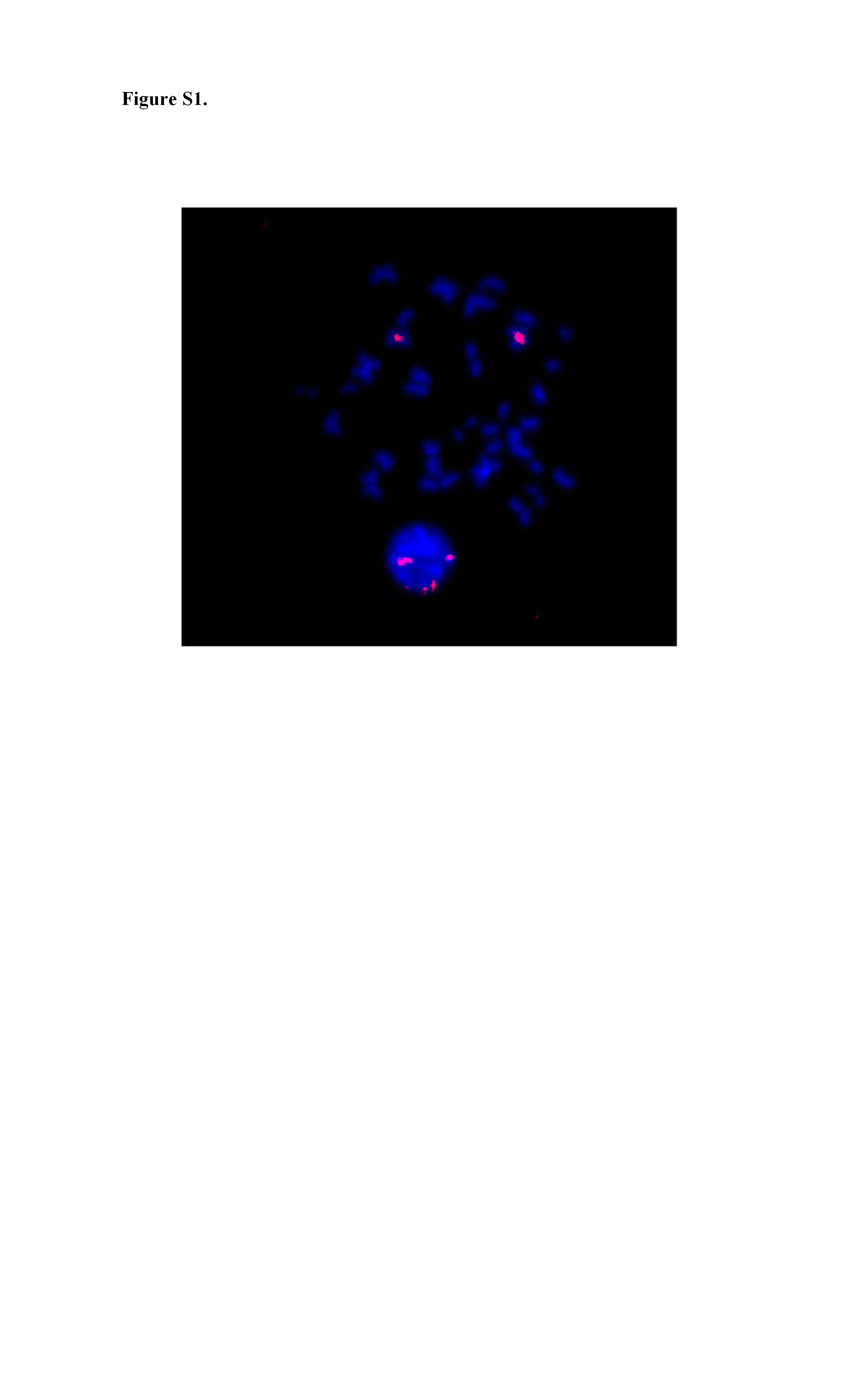

Supplement: Figure S1 — Examples of FISH using a centromeric probe specific for chromosome 7 (7cen) on patient no. 1. Two red signals are clearly visible in the metaphase, whereas three fluorescent signals are present in the nucleus in the same sample. (TIF) [file pone.0020607.s001.tif]
