# Supplementary material for: Genomic Imbalances Are Confined to Non-Proliferating Cells in Paediatric Patients with Acute Myeloid Leukaemia and a Normal or Incomplete Karyotype
Source: PLoS One. 2011 Jun 9;6(6):e20607. doi: 10.1371/journal.pone.0020607 (PMC3111408; doi:10.1371/journal.pone.0020607)
Supplement: Table S1 — Using a one-sided hypothesis test, with a null hypothesis that p1 = p2, and an alternative hypothesis that p1<p2, at 99% confidence, the critical test statistic is 2.33. Since the test statistics above are all greater than 2.33, then we can reject the null hypothesis in all cases. Note: it is often recommended that this test only be applied when both the number of successes and the number of failures is at least 5 in both populations. Although this is strictly not satisfied in population 2 above, it is satisfied for the cell population as a whole. Also, if the number of failures in population 2 (i.e. the number of Ki-67 positive cells with 3 signals) was 5 for all patients (a result which is clearly not as significant as the result above), then the result would still be statistically significant at 99% confidence for all patients except patient 5. For patient 5 it would be statistically significant at 98% confidence. (DOC) [file pone.0020607.s002.doc]

| patient no. | **n_1_** (no. of nuclei with two signals) | **n_2_** (no. of nuclei with three signals) | **p_1_** (proportion of **n_1_** which are Ki-67 negative) | **p_2_** (proportion of **n_2_** which are Ki-67 negative) | sample proportion | test statistic (N(0,1) under null hypothesis) |
| --- | --- | --- | --- | --- | --- | --- |
| 1 | 228 | 121 | 0.746 | 0.975 | 0.825 | 5.375 |
| 2 | 130 | 73 | 0.754 | 0.932 | 0.818 | 3.146 |
| 5 | 155 | 40 | 0.710 | 0.950 | 0.759 | 3.168 |
| 11 | 79 | 101 | 0.696 | 1.000 | 0.867 | 5.950 |
| Total cell population | 592 | 335 | 0.731 | 0.970 | 0.818 | 9.044 |

**Table S1.** Application of the hypothesis test applied to our datasets of four patients.
